# Food Allergen Quantitative Risk Assessment at a Crossroads: A Critical Evaluation of Laboratory Performance for Quantifying Total Egg and Milk Protein in Cookies

**DOI:** 10.3390/foods14060957

**Published:** 2025-03-11

**Authors:** Elena Cubero-Leon, Jørgen Nørgaard, Pieter Dehouck, Piotr Robouch

**Affiliations:** European Commission, Joint Research Centre (JRC), 2440 Geel, Belgium

**Keywords:** proficiency testing, quantitative risk assessment, total protein content, immunoassays, allergens, milk protein, egg protein

## Abstract

The accurate quantification of food allergens is crucial for ensuring consumer safety and compliance with regulatory requirements. A proficiency test (PT) was organised to evaluate the performance of laboratories in quantifying total egg and total milk protein in cookies. The PT involved 20 laboratories, which reported results using mainly commercial ELISA kits and liquid chromatography–tandem mass spectrometry (LC-MS/MS). The findings indicate a satisfactory performance for milk protein determination among the majority of participant laboratories. However, the quantification of egg proteins in heated products remains a challenge, with most laboratories reporting results significantly below the reference value. Several potential factors contributing to this challenge are discussed, including the denaturation of egg proteins during heat treatment, differences in extraction strategies and the antibodies used in ELISA kits, and the lack of standardised methods and conversion factors for LC-MS/MS analysis. These findings underscore the importance of regular PT exercises to evaluate laboratory performance and ensure compliance with WHO/FAO recommendations. The results of this study aim to guide the development of improved analytical methods and strategies for ensuring the accurate quantification of food allergens.

## 1. Introduction

Allergens may be introduced into food products intentionally as ingredients or as a result of cross-contact where allergens are transferred to a food not originally containing them (unintended allergen presence, UAP). While the legislative framework is different for many regions/countries, EU Member states and many other countries have a priority list of allergens that need to be declared in ingredient lists when added intentionally. However, regulatory frameworks on UAP and the application of precautionary allergen labelling (PAL), used to communicate potential risks to food-allergic individuals, are generally lacking [1]. Hence, there is diverse and inconsistent use of PAL by the industry, making it less effective in protecting people with food allergies [2].

To address the issue of PAL, a joint Food and Agriculture Organization of the United Nations (FAO) and World Health Organization (WHO) expert consultation on Risk Assessment of Food Allergens was convened in 2019 [3]. This consultation focusses on a recommended priority list of allergenic foods based on risk assessment, recommended reference doses (RfDs) to inform management of UAP of priority allergens in food, a PAL framework, and the exemption framework. RfDs are expressed as mg of total protein from the allergenic food and “reflect a range of exposure without appreciable health risk” [3]. The level of exposure to a UAP in a food will also depend on how much food is consumed. The expert consultation reports provide action levels. When a product is above such a level, it is required to have PAL for each priority allergen, taking into account both the RfDs and different intake ranges.

The outputs of this consultation are currently being considered by the Codex Committee on Food Labelling (CCFL) for implementation into global food standards. Although the guidance is still under review, there has already been an immediate impact of the recommendations in several countries. Examples include the Netherlands, which has introduced a new policy for UAP and PAL based on the FAO/WHO recommendations [4], and Belgium, which has recently updated their RfDs for allergens to align with the FAO/WHO recommendations [5]. Australia and New Zealand have adopted the FAO/WHO RfDs based on ED05 values through the VITAL program [6]. Meanwhile, the UK is seeking a board view on the approach to PAL in the context of the international Codex guidance [7], and Germany has published ‘assessment values’ based on the FAO/WHO RfDs [8], which are highly recommended but not mandatory.

While advancements in harmonised food allergen risk assessment and the use of PAL are progressing, their successful implementation requires analytical workflows capable of producing reliable and comparable measurement results [9]. The comparability of measurement results in food allergen analysis is hindered by the different quantification parameters targeted by existing analytical procedures. As a first step towards obtaining comparable data fit for risk assessment, the analytical community has agreed on a common measurand, “mg total protein of the allergenic food per kg of food analysed” [3,10]. This is how laboratories should express their analytical test results regardless of the methodology being used. Therefore, what is actually measured (analyte) by the specific measurement procedure must be converted into the common measurand, which requires a mathematical relationship between the parameters measured and the agreed measurand. Additionally, existing analytical methodologies need to be evaluated to ensure they can effectively monitor the compliance of food products with the proposed RfDs. In this respect, the Codex Committee of Methods of Analysis and Sampling (CCMAS) is supporting the Codex work by assessing whether existing methodologies meet the necessary performance criteria to detect and quantify food allergens in line with the consultation’s recommendations [11].

In an effort to harmonise EU food allergen measurements, the European Network for Food Allergen Detection Laboratories (ENFADL), hosted by the Joint Research Centre (JRC) of the European Commission, conducted a feasibility proficiency testing round (PT) in 2018 [12]. The PT focussed on the determination of the “mass fraction of total cow milk protein in a baked cookie”. The laboratories were required to report their results in this common measurand suitable for risk assessment. Unlike other commercial allergen analysis PT schemes, which provide kit-specific consensus values and accept various reporting units, this PT utilised a tailor-made test material with an assigned value determined by a reference method traceable to the International System of Units (SI) [13]. This approach allowed an assessment of the equivalence of measurement results across laboratories, regardless of the specific methods employed. The results showed divergent results among different ELISA kits. Laboratories and test kit manufacturers were invited to a workshop to discuss the results, which were later published by Cordeiro et al. [12]. The JRC has since organised a second PT to assess laboratory performance for the determination of total egg and milk protein in cookies. The findings of this exercise, detailed in this manuscript, will provide valuable insights into laboratory preparedness and method suitability for determining total egg and milk protein content, considering recent advancements in food allergen risk assessment.

## 2. Material and Methods

### 2.1. Preparation of the Test Item

Incurred test cookies were prepared by following the procedure described by Cordeiro et al. [12]. In brief, fat (Vitalite dairy free, Saputo Cheese USA Inc., Lincolnshire, IL, USA), sugar (Raffinerie Tirlemontoise s.a., Tirlemont, Belgium), and flour (Everyday, Colruyt N.V./S.A., Halle, Belgium) were mixed to form a dough, and skim milk powder (BIOSERVICE Zach GmBH, Schrems, Austria), whole egg powder (ProteinVital, Brandsvital, Vienna, Austria), ammonium bicarbonate (Merck KGaA, Darmstadt, Germany) and salt were added to this mixture. Thirty-gram balls of dough were formed, flattened to a thickness of 1.5 mm, cut into circular shapes, and placed on greaseproof paper to bake at 155 °C for 14 min. Following baking, the cookies were ground twice, first using a Retsch mill (ZM200, Retsch GmBH & Co, Haan, Germany) and then an Ultraturrax (IKA, ULTRA-TURRAX T10 Basic, Staufen, Germany) mixer in liquid nitrogen to produce a fine powder. The resulting powder was then portioned into 5 g samples (test items), which were vacuum-packed and stored at −4 °C.

### 2.2. Homogeneity and Stability

To assess the homogeneity and stability of the test material, two commercially available enzyme-linked immunosorbent assay (ELISA) kits were used: β-lactoglobulin ELISA Kit II and Egg (ovalbumin) ELISA Kit II (both from Morinaga Institute of Biological Science, Inc., Yokohama-Shi, Japan, Cat. No. M2112 and M2111). For homogeneity, ten sachets were randomly selected and analysed in duplicate. The results were evaluated according to Clause B.2.2 of ISO 13528:2022 [14], by comparing the between-sample standard deviation (*s*_s_) with the standard deviation for proficiency assessment (*σ*_pt_). The test material proved to be adequately homogeneous for the marker proteins investigated (β-lactoglobulin and ovalbumin) at a sample intake of 1 g (*s*_s_ ≤ 0.3 *σ*_pt_). The contribution from homogeneity (*u*_hom_) to the standard uncertainty of the assigned value (*u*(*x*_pt_)) was determined in accordance with ISO Guide 35:2017 [15].

The assessment of stability was performed by keeping 3 sachets of the test material at +4 °C and at a reference temperature of −18 °C for the duration of the PT (from sample dispatch to reporting deadline). Duplicate results from 2 sachets at each temperature were analysed, and the average of the 4 results for each temperature was calculated. The stability of the test material was verified according to Clause B.5.1 of ISO 13528:2023 [14], by comparing the average of the measurements obtained at the two temperatures. Having met the criteria, it was confirmed the material was adequately stable throughout the PT period. Consequently, the uncertainty contribution due to stability (*u*_stab_) was set to zero.

### 2.3. Value Assignment and Uncertainty Estimation

#### 2.3.1. Milk

The assigned value (*x*_pt_) of the mass fraction of total cow milk protein in the baked cookie was established using the method described by Martinez-Esteso et al. [13]. The process involved extracting proteins from three test units of test material, randomly selected, and using a buffer containing urea, ammonium bicarbonate, and dithiothreitol. An aliquot of the extract was then spiked with a mixture of stable-isotope-labelled isotopologues (ranging from six to twenty amino acids in length) of eleven target peptides, from five proteins (α_S1_-casein (CASA1), α_S2_-casein (CASA2), β-casein (CASB), κ-casein (CASK), and β-lactoglobulin (LACB)). The labelled isotopologues were purchased from JPT (Peptide Technologies GmbH, Berlin, Germany). Each test unit was subjected to two separate digestions and clean-up steps using 200 mg HyperSep™ C18 columns (Thermo Scientific, Biopolymers, Ulm, Germany), following the protocol described in detail by Martinez-Esteso et al. [13]. The resulting injection solution was then analysed by liquid chromatography–tandem mass spectrometry (LC-MS/MS) to determine the total protein content.

The method provided results traceable to the SI through calibrants consisting of synthetic peptides (analogues of the target peptides), ranging from six to twenty amino acids in length, whose purity was determined by amino acid analysis following the procedure described by Muñoz et al. [16]. Synthetic peptides were purchased from Thermo Fisher Scientific (Waltham, MA, USA). A calibration curve was prepared by adding the calibrants containing the eleven synthetic peptides [13] in a blank baked cookie free of milk. A matrix blank free of these peptides was run to confirm the absence of significant analyte signals.

The analysis was conducted using an LC-MS/MS system, comprising a Waters Acquity M class chromatography system coupled to a Waters Xevo TQ-XS mass spectrometer (Waters, Manchester, UK). Chromatographic separation was achieved using a 150 µm x 100 mm Peptide BEH C18 column with an integrated ESI emitter, as part of an IonKey-based system (Waters, Manchester, UK). The measurements were performed in Multiple Reaction Monitoring (MRM) mode, with a dwell time of 40 ms and optimised collision energies for each peptide. A 2 µL sample volume was injected at a flow rate of 2 µL/min, with the following gradient program: 0–5% solvent B for 0.5 min, 5–25% solvent B over 17.5 min, and 25–40% solvent B over 4.5 min. The mobile phases consisted of water/formic acid (999:1 v/v; solvent A) and acetonitrile/formic acid (999:1 *v*/*v*; solvent B).

An assigned value (*x*_pt_) of 20.9 mg kg^−1^ was obtained and used to assess the laboratory performance. The standard uncertainty of the assigned value (*u*(*x*_pt_)) was estimated by combining the standard measurement uncertainty of the characterisation of the material (*u*_char_), with the standard uncertainty contributions due to homogeneity and stability (*u*_hom_ and *u*_stab_) [14]. The uncertainty contribution due to characterisation (*u*_char_) was estimated by combining the uncertainties of the calculated amount of proteotypic peptide in test solution, the protein mass fraction in the test material, and the mass fraction of the total cow milk protein, as described by Breidbach et al. [17]. The uncertainty contribution due to inhomogeneity (*u*_hom_) of 6% was derived from the homogeneity study, while the uncertainty contribution due to stability (*u*_stab_) was set to zero. An expanded uncertainty *U*(*x*_pt_) of 3.3 mg kg^−1^ was calculated using a coverage factor of 2.

#### 2.3.2. Egg

A similar experimental approach was used to quantify the mass fraction of total hen egg protein in the baked cookie. Proteins were extracted from the test material and fortified with stable-isotope-labelled isotopologues of the target peptides and then digested and cleaned up. The procedure is detailed in Martinez-Esteso et al. [13]. The resulting injection solution was analysed with an LC-MS/MS system as described earlier, targeting only two specific peptide sequences derived from ovalbumin in egg white: ELINSWVESQTNGIIR (ELI) and GGLEPINFQTAADQAR (GGL).

A calibration curve was prepared from an egg-free baked cookie that was fortified with a mixed reference solution containing synthetic peptides (analogues of the target peptides), whose purity was determined by amino acid analysis following the procedure described by Muñoz et al. [16]. Synthetic peptides were purchased from Thermo Fisher Scientific (Waltham, MA, USA). To assign the value, eight test units of the test material were randomly selected and analysed by two operators over a period of two days. Each operator performed two separate extractions of two test units per day, with each unit undergoing duplicate digestions and preparations. A matrix blank, free of the two peptides, was analysed to confirm the absence of significant analyte signals. The mass fraction of total hen egg protein was calculated from the measured mass fraction of ovalbumin, using a conversion factor of 0.445 ± 0.045 (*u*, *k* = 1) derived from the literature [18,19]. The resulting informative value of 10 mg kg^−1^ was used to calculate the relative difference (*D%*) and evaluate laboratory performance.

### 2.4. Proficiency Test

ENFADL member laboratories and a few Belgian official control laboratories were invited to participate in this study. They registered through an in-house-developed online tool for inter-laboratory comparisons (MILC). The test items were dispatched on dry ice and had to be stored in a dark place at 4 °C ± 2 °C upon arrival. Detailed instructions were provided, and the laboratories were advised to use a method that resembled as closely as possible the one they use for routine analysis. Independently of the method used, the laboratories were asked to report their results in the common measurand as mass fraction of total hen egg protein in cookie (mg kg^−1^) and mass fraction of total cow milk protein in cookie (mg kg^−1^). The results were required to be submitted within 8 weeks after dispatch using the online MILC reporting platform. Additional information was requested through a questionnaire provided via EUSurvey.

For the determination of total cow milk protein, the *σ*_pt_ was set to 25% of the assigned value, based on expert judgment. As the *u*(*x*_pt_) exceeded 0.3 *σ*_pt_, the laboratory performance was assessed in terms of the *z*′ (z prime) score according to ISO 13528:2022 [14]. In contrast, the performance of laboratories in determining total hen egg protein was assessed using *D%*, which estimates the deviation between the reported result and the assigned value.

The following equations were used for the evaluation mentioned earlier:

(1) $$\begin{array}{cc} D\% & =100\;\frac{x_{i}-x_{pt}}{x_{pt}}\;\mathrm{and}\\ z_{i}^{\prime } & =\frac{x_{i\;}-x_{pt}}{\sqrt{\sigma_{pt}^{2}+u^{2}(x_{pt})}} \end{array}$$

The outcome of this PT was discussed with the participants during the annual ENFADL workshop and subsequently documented in a confidential report [20].

## 3. Results and Discussion

### 3.1. Milk

Twenty laboratories submitted a total of 22 results, with two laboratories providing results from two different measurement procedures. Table 1 details the analytical methods used by the laboratories and the targeted analytes/proteins, where known. Two laboratories (L002 and L006a) reported truncated (“higher-than”) values. Two laboratories (L013 and L014) did not comply with the requirements, as they reported casein instead of total cow milk protein; therefore, their performance was not assessed. Most laboratories, 16 out of the 22, used commercial ELISA test kits from various manufacturers. One of the laboratories using LC-MS/MS reported a “higher-than” value and stated in the questionnaire that their method was qualitative and not within the scope of their accreditation.

**Figure 1:**
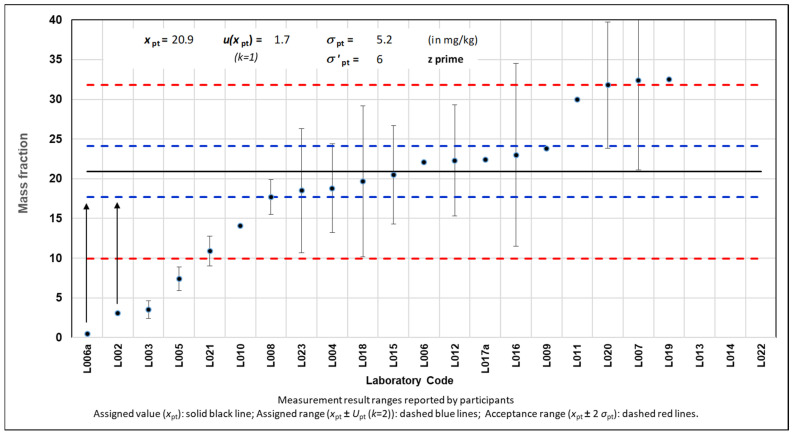
Mass fraction of total cow milk protein in cookies, in mg kg^−1^, as reported by participating laboratories. Arrows represent truncated values.

Figure 1 illustrates the reported results including their expanded measurement uncertainties (error bars) where available. Most laboratories (80%) demonstrated satisfactory performance according to the *z′* score (|*z′*| ≤ 2.0); see the results falling within the acceptable range area, represented with red dashed lines, in Figure 1. This confirms the effective performance of the laboratories and commercial test kits used in this PT for determining total milk protein in cookies. This conclusion is further supported by the significant overlap observed between the assigned range (20.9 ± 3.3 (*k* = 2) mg kg^−1^) and the robust range calculated using Algorithm A [14] (20.9 ± 9.5 (*k* = 2) mg kg^−1^). Only laboratory L003 showed unsatisfactory performance (|*z′*| ≥ 3.0).

In contrast to the similar PT conducted in 2018 by the JRC [12], which reported an average ELISA bias of −40% in a baked cookie material, the present PT demonstrated a significantly reduced bias of −4% (as calculated by comparing the robust mean and the reference value). The authors of the previous study emphasised the need for improved standard operating procedures (SOPs) to ensure clear interpretation of their applicability and the need for transparent metrological traceability of their calibrants. Following these findings, some test kit manufacturers released new SOPs, likely validating the nature of their calibrants. This development was further confirmed during the 2022 ENFADL annual workshop, where ENFADL member laboratories reported revalidating their ELISA methods based on the new ELISA test kits. These changes may have contributed to the significant improvement in performance observed in the current PT round.

It is worth noticing that only 12 laboratories reported measurement uncertainties, and they were mainly derived from their single-laboratory validation study or the standard deviation of replicate measurements.

The risk of exposure to a specific allergen by UAP does not only depend on the amount of the UAP in that food product but also on the quantity of food consumed. Recent WHO/FAO consultation reports [3] provide comprehensive tables of action levels for each priority food allergen taking into account both, the RfDs and different intake ranges. Action levels represent concentrations of UAP above which a response may be required, such as implementation of PAL, recall decisions, or trade rejections. For milk in cookies, the recommended action level corresponds to 50 mg of total protein from the allergenic food per kilogram of food taking into account the food intake estimates presented in the report. Notably, the baked cookie test material used in this PT exercise had a concentration 2.4 times lower than the recommended action level. Furthermore, WHO/FAO meeting reports [3] specify that the limit of quantification (LOQ) of any analytical method used for this purpose should be around three-fold lower than the action level, to account for variability and ensure that the analytical result is truly above or below the action level. The LOQ estimates of all laboratories that reported method performance characteristics in the questionnaire met this criterion.

These findings provide strong evidence that the majority of laboratories are capable of accurately monitoring mass fractions of total cow milk protein in cookies, thereby supporting the new WHO/FAO recommendations.

### 3.2. Egg

**Figure 2:**
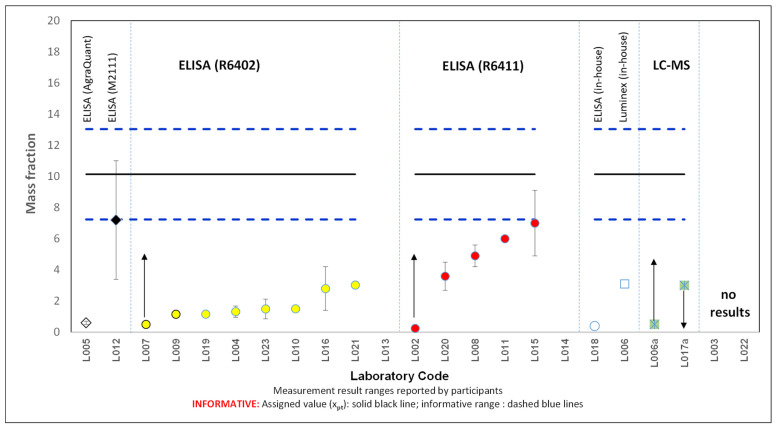
Mass fraction of total hen egg protein in cookies, in mg kg^−1^, as reported by participating laboratories. Arrows represent truncated values. Different symbols represent different analytical methodologies (as specified above the upper dashed blue line).

Nineteen laboratories submitted a total of 21 results, with two laboratories providing results from two different measurement procedures. Table 1 provides details about the analytical methods used by laboratories and targeted analytes/proteins where known. Four laboratories provided truncated values (L017a: “lower-than”; L002, L006a, L007: “higher-than”). Two laboratories (L013 and L014) did not comply with the requirements and reported egg mass instead of total hen egg protein; therefore, their performance was not assessed. Two laboratories used LC-MS/MS and reported truncated values (“lower-than” and “higher-than”). Laboratory L006a stated in the questionnaire that their method is still under development, while L017a detected a level below their LOD of 3 mg kg^−1^. Most laboratories’ results, 17 out of the 21, were obtained using different commercial ELISA test kits from various manufacturers. Figure 2 indicates the test kits used and the reported results, including their expanded measurement uncertainties (error bars), where known.

The results ranged from a −96% to a −31% percent difference (*D%*) compared to the reference value of 10 mg kg^−1^. Most laboratories reported results significantly below this reference value. Only two laboratories (L012 using Morinaga Egg (Ovalbumin) ELISA KIT II Cat. No. M2111 and L015 using R-Biopharm RIDASCREEN Egg test kit, Cat. No. R6411) achieved *D%* values of around −30%. This poor overall performance is further substantiated by the significantly lower robust mean consensus value of 3 mg kg^−1^, calculated from the reported results using Algorithm A [14].

Laboratories that used R-Biopharm RIDASCREEN Fast Ei/Egg Protein (R6402) reported lower values compared to those using RIDASCREEN Egg (R6411) (Figure 2). This discrepancy can be attributed to the differences stated in the protocol between the two kits. Specifically, the R6402 protocol notes that proteins in processed foods may be altered or fragmented, affecting recovery and assay results, particularly in heat-treated samples where egg proteins denature and become unrecognisable by the ELISA antibody. R-Biopharm recommends in those instances to use the R6411 Kit, which includes a specific extraction buffer with an additive designed for processed and heated samples like noodles and cookies.

These results highlight the challenge of quantifying egg proteins in heated products. It is known that egg white proteins, when exposed to high temperatures, can undergo denaturation or chemical modifications, leading to reduced solubility and antibody recognition affecting the detection and quantification by different ELISAs [18,21,22,23]. Ovomucoid, which is also one of the targets of the R-Biopharm test kits R6402 and R6411 used by the laboratories, has been found to be more resistant to thermal denaturation than ovalbumin [22], but a decrease in its solubility in the presence of wheat and prolonged heat has been reported [24]. This decrease in extractability may be attributed to heat-induced protein aggregation through the formation of intermolecular disulphide bonds [25]. The Morinaga (Ovalbumin) Egg kit (Cat. No. M2111) incorporates a surfactant, SDS, and a reducing agent, 2-mercaptoethanol, into its extraction buffer, which enhances the solubilisation of proteins in processed food samples, thereby improving extraction efficiency [26]. Parker et al. [18], Faeste et al. [27], and Gomaa et al. [23] have found that the Morinaga kit performed better in thermally processed samples, showing higher recoveries than other commercial test kits. They also state that these differences are most probably due to the different extraction strategies used (denaturing–reduction conditions), resulting in higher extractions. It is worth noting that the RIDASCREEN Egg kit (R6411) has adopted a similar approach incorporating a sulphite-containing extracting agent into their extraction buffer that is patented by Morinaga & Co as stated in the kit manual.

Underestimation of total egg protein content may also occur due to alterations in the immunoreactivity of residual proteins following heat treatments rather than differences in protein extraction efficiency. Fu et al. [22] showed that dry heating above 177 °C for 10 min can alter the immunoreactivity of egg white proteins, thereby affecting the accuracy of protein quantification by commercial ELISA test kits. The validation report for the RIDASCREEN Egg kit (R6411) also shows low recovery rates of around 50% for cookies containing whole egg powder baked at a high temperature (150 °C) and extracted using the recommended reagent for processed samples, aligning with the results obtained in this study. The Morinaga test kit uses an antibody prepared through immunisation with an antigen denatured with SDS and 2-mercaptoethanol. It is therefore able to recognise the denatured form of the protein compatible with the extraction conditions, which otherwise loses its binding ability to an anti-native ovalbumin antibody [26].

A recent paper by Smits et al. [28] similarly stated through a personal communication with Allergen Consultancy that the detection of egg in crisp cookies or rusk can be challenging according to experienced users. In this study, they were able to obtain good recoveries in spiked material, but in incurred material, the egg protein levels measured by different test kits differed substantially. These studies underscore the challenges of using ELISA kits to quantify egg proteins in heated products and suggest that improvements in protein solubilisation strategies for sample extraction protocols, as well as the development of antibodies with enhanced specificity to heat-treated protein markers, can significantly improve the analytical detectability of the ELISA tests in this type of product.

A few laboratories reported “qualitative” (truncated) results for total cow milk and egg proteins, mainly derived from LC-MS/MS measurements. This may be due to the challenges associated with developing quantitative LC-MS/MS methodologies. While synthetic peptides can serve as calibrants for estimating the concentrations of specific peptides, a mathematical conversion is required to translate these values into the total egg protein content, the relevant measurand for quantitative risk assessment. This approach has not been widely adopted, apart from a few recent examples for egg, milk, and peanut [13,18,19,29,30,31,32], primarily due to the lack of standardised methods and established conversion factors necessary for these calculations.

## 4. Conclusions

The results of this PT demonstrate that the majority of laboratories are capable of accurately monitoring mass fractions of total cow milk protein in cookies, thereby supporting the new WHO/FAO recommendations. Significant progress in laboratory performance for milk protein determination in cookies was observed compared to the previous PT. However, the quantification of egg proteins in heated products remains a challenge, with most laboratories reporting results significantly below the reference value. The adoption of ELISA kits with optimised extraction strategies and antibodies designed for enhanced specificity to heat-treated protein markers may improve the analytical detectability of these tests.

The results of this PT highlights that, despite progress in the quantification and reporting of food allergen measurements, challenges persist in estimating measurement uncertainties and reporting results as total protein from the allergenic ingredient in food. To address these issues, the European Network for Food Allergen Detection Laboratories (ENFADL) has established two working groups. The first group focusses on developing guidelines and best practices for determining realistic measurement uncertainty in food allergen analysis. The second group is dedicated to establishing agreed conversion factors for translating measured values into the defined measurand.

Assigning a reference value for test items and standardising the reporting unit for laboratory results ensures comparability and facilitates the evaluation of laboratory performance. Regular PT exercises are essential for assessing this performance and ensuring compliance with WHO/FAO recommendations.

## Figures and Tables

**Table 1:** Analytical methods used by laboratories and proteins/peptides targeted by the method.

Analytical Method	Laboratories	Target Protein/Peptide
**Milk**		
LC-MS/MS	L006a	Casein (FFVAPFPEVFGK, NAVPITPTLNR) and β-lactoglobulin (VLVLDTDYK)
LC-MS/MS	L017a	Casein (FFVAPFPEVFGK) and β-lactoglobulin (LSFNPTQLEEQCHI)
ELISA: R-Biopharm RIDASCREEN Fast Milk (Cat. No. R4652)	L004, L007, L009, L010, L011, L016, L019, L020, L023	Casein and β-lactoglobulin
ELISA: R-Biopharm RIDASCREEN Fast Casein (Cat. No. R4612)	L002, L008, L013, L014	Casein
ELISA: Morinaga Casein ELISA Kit II (Cat. No. M2113)	L012, L015	Casein
ELISA: Veratox Total Milk (Cat. No. 8470)	L021	Multiple milk proteins (identity undisclosed)
ELISA: In-house-developed	L018	Casein
ELISA: Unidentified	L003, L005, L006	Undisclosed
**Egg**		
LC-MS/MS	L006a	Ovalbumin (peptide sequence undisclosed)
LC-MS/MS	L017a	Ovalbumin (GGLEPINFQTAADQAR) and Vitellogenin-1 (YLLDLLPAAASHR)
RIDASCREEN Fast Ei/Egg Protein (Cat. No. R6402)	L004, L007, L009, L010, L013, L016, L019, L021, L023	Ovalbumin and ovomucoid
ELISA: R-Biopharm RIDASCREEN Egg (Cat. No. R6411)	L002, L008, L011, L014, L015, L020	Ovalbumin and ovomucoid
Morinaga Egg (Ovalbumin) ELISA KIT (Cat. No. M2111)	L012	Ovalbumin
Romer Labs AgraQuant^®^ Egg White (Cat. No. 10002205)	L005	Egg white proteins (identity undisclosed)
ELISA: In-house-developed	L018	Ovomucoid
Luminex: In-house-developed	L006	Undisclosed

## Data Availability

The original contributions presented in this study are included in the article. Further inquiries can be directed to the corresponding author.
